# Associations between quality of life in communication and sociodemographic and clinical aspects of Parkinson's disease

**DOI:** 10.1590/2317-1782/e20240298en

**Published:** 2025-10-13

**Authors:** Karoline de Abreu Silveira, Guilherme Briczinski de Souza, Camila Muratt Carpenedo, Carlos Roberto de Mello Rieder, Bárbara Costa Beber

**Affiliations:** 1 Curso de Fonoaudiologia, Universidade Federal de Ciências da Saúde de Porto Alegre – UFCSPA - Porto Alegre (RS), Brasil.; 2 Programa de Pós-Graduação em Ciências da Reabilitação, Universidade Federal de Ciências da Saúde de Porto Alegre – UFCSPA - Porto Alegre (RS), Brasil.; 3 Department of Internal Medicine, Universidade Federal de Ciências da Saúde de Porto Alegre – UFCSPA - Porto Alegre (RS), Brasil.; 4 Departamento de Fonoaudiologia, Universidade Federal de Ciências da Saúde de Porto Alegre – UFCSPA - Porto Alegre (RS), Brasil.

**Keywords:** Parkinson Disease, Quality of Life, Communication, Signs and Symptoms, Speech Disorders

## Abstract

**Purpose:**

To investigate the correlation between quality of life in communication among people with Parkinson's disease and clinical and sociodemographic variables.

**Methods:**

Cross-sectional study that analyzed sociodemographic variables - such as sex, age, education and ethnicity - and clinical variables, including age at onset of symptoms, time since PD diagnosis, initial symptom, equivalent daily dose of levodopa, motor impairment, stage of the disease, cognition, mood disorders and quality of life. The correlation test and multiple linear regression model were used for statistical analysis.

**Results:**

The sample consisted of 34 individuals, with a mean age of 62.42 years (±12.21), mostly male (61.76%) and with a mean score of 20.09 (±17.78) in the communication item of the PDQ-39. A significant correlation was observed between communication and disease duration, depression and anxiety levels, activities of daily living, emotional well-being, stigma, social support, cognition, bodily discomfort, disease stage (Hoehn & Yahr), as well as motor and non-motor aspects and the total score of the PDQ-39 scale. This indicates that communication is affected by many areas of life and by the progression of the disease. When analyzed together (multiple linear regression model), activities of daily living and non-motor aspects (such as emotional and cognitive problems) are the main predictors of quality of life related to communication.

**Conclusion:**

Communication in individuals with PD is influenced by several factors related to the progression of the disease. Among them, activities of daily living and non-motor aspects stand out as the main influences on quality of life in communication.

## INTRODUCTION

Parkinson's disease (PD) is a rapidly progressive neurodegenerative condition with a variety of causes and clinical presentations. PD is the second most prevalent neurodegenerative disease, with a global prevalence of 6 million people. This movement disorder is characterized by bradykinesia, tremor, rigidity, and postural instability^(1,2)^.

In addition to the classic motor symptoms, speech disorders may also be present, affecting around 89% of people with PD. Hypokinetic dysarthria is a term used to describe the speech disorder in PD, which can appear in the later stages of the disease as well as in its early phase. Hypokinetic dysarthria is characterized by changes in the amplitude, speed, strength, precision, or tone of the movements required for speech production, and other components, such as phonation, resonance, breathing, and prosody, may also be impaired. Hypokinetic dysarthria becomes more severe as the disease progresses, causing significant difficulties in communicating^(2-4)^.

However, changes in communication in PD go beyond hypokinetic dysarthria. Language changes may be present in the early stages of the disease. The literature indicates that individuals with PD have difficulties with verbal and nominal inflection, deficits in the construction and understanding of complex sentences, difficulties in lexical access and naming, as well as impairments in the interpretation of irony, sarcasm and contextual clues, which compromises the adequate understanding of communicative interactions^(5)^.

These communication difficulties do not occur in isolation in PD. Cognitive deficits are commonly observed, with a heterogeneous cognitive profile, which may include memory impairments, executive dysfunction, and visuospatial difficulties^(2)^. These cognitive alterations are closely related to linguistic deficits, further impacting communication. In addition to cognitive aspects, symptoms of depression and anxiety are also prevalent in PD^(2)^. However, mood changes can often go unnoticed, and it remains unclear how they interact with motor symptoms to influence communication in individuals with the disease.

Quality of life is a broad measure that refers to an individual's perception of their well-being and encompasses various aspects of life, such as physical and mental health, education, safety, housing, employment, environment, social relationships, culture, leisure, and other factors that contribute to a person's satisfaction and happiness^(6)^. To assess the quality of life in PD patients, several scales have been proposed, with the PDQ-39 being one of the most widely used worldwide. This scale has been translated, culturally adapted, and validated into 13 different languages, providing a comprehensive tool for evaluating the impact of PD on various aspects of life. Studies indicate that PD interferes with people's autonomy and independence, with motor, cognitive, and emotional aspects being the main factors^(7-12)^.

Communication is an essential element for autonomy and quality of life, and its influence on activities of daily living is significant. The communication difficulties associated with PD reduce speech intelligibility and understanding of inferences, making social interactions and daily activities challenging. This may impact everything from simple tasks, such as shopping and talking on the phone, to more complex situations, such as reporting symptoms to health professionals or expressing emotions and needs. Limitations in communication affect not only individual functionality, but also interpersonal relationships, and can lead to greater dependence on others to carry out activities, social isolation and compromised mental health.

Although the literature presents robust studies on quality of life in various aspects of the lives of people with PD, there are specific domains of quality of life that can be further explored, such as communication-related quality of life in people with PD. In this context, this study aimed to investigate the correlation between quality of life in communication of people with PD and clinical and sociodemographic variables.

## METHODS

This is a cross-sectional study approved by the ethics and research committee of the Irmandade Santa Casa de Misericórdia de Porto Alegre (ISCMPA) under opinion number 3,258,886. All individuals who participated in the study signed the informed consent form.

This study included individuals with PD, who were followed between June 2019 and May 2020 at the Movement Disorders outpatient clinic of the Sistema Único de Saúde (SUS) at ISCMPA, as well as at health insurance clinics. All individuals were examined by a single physician, a specialist in movement disorders, according to the criteria for idiopathic PD by the PD Society UK Brain Bank^(13)^. Individuals with a medical diagnosis of atypical or secondary parkinsonian syndrome, individuals who refused to participate in the research and those with a medical diagnosis of dementia were excluded from the sample.

For this study, the sociodemographic variables collected were sex, age, education and ethnicity. And the clinical variables were age at onset of symptoms, time since PD diagnosis, initial symptom, equivalent daily dose of levodopa, motor impairment, stage of the disease, cognition, mood disorders and quality of life.

Data collection was performed in a single session by a neurologist. The time for each assessment varied from one to two hours and all patients were in an “alert” behavioral state. Sociodemographic and clinical data (age, sex, ethnicity, education, age at onset of symptoms, initial symptoms, time since PD diagnosis and daily dose of levodopa) were collected through data from medical records and by completing a questionnaire developed for the study and answered by the individual or by a family member/caregiver, if assistance was needed.

In order to obtain a standardization of the data on the equivalent daily dose of levodopa (LEDD), the calculation from a previous study was used^(14)^, which sought to provide a formula to express the dose intensity of different antiparkinsonian drug regimens on a single scale.

The Unified Parkinson's Disease Rating Scale (MDS-UPDRS) was used to grade motor impairment^(15)^. This scale assesses the signs, symptoms and specific activities of patients through self-reporting and clinical observation, and allows monitoring of disease progression and the effectiveness of drug treatment. It consists of 42 items, divided into four parts: mental activity, behavior and mood; activities of daily living; motor exploration and complications of drug therapy. The score for each item ranges from zero to four, with the maximum value indicating greater impairment due to the disease and the minimum normality.

In order to classify the stage of PD in which the individuals were, the Hoehn & Yahr (HY) scale was used^(16)^. HY is a scale that classifies PD severity into five stages and encompasses global measures of disability level. Patients classified in stages I, II, and III have mild to moderate disability, while those in stages IV and V have more severe disability.

The Montreal Cognitive Assessment (MoCA) was used to assess participants' cognition^(17)^. The MoCA is a cognitive screening instrument that assesses executive function, language, orientation, attention, memory and constructive praxis. The maximum score is 30 points, and the cutoff point for normality is a score greater than or equal to 26.

In order to identify mood disorders, the Beck Anxiety Inventory (BAI) and the Beck Depression Inventory (BDI) were used^(18,19)^. The BAI consists of 21 items describing common symptoms in anxiety disorders, and the individual must indicate between four points (zero - absent and four - severe). The items added together result in within the minimum limit (zero to 10 points), mild anxiety (11 to 19 points), moderate anxiety (20 to 30 points), severe anxiety (31 to 63 points). The BDI, through self-report, assesses the intensity of depressive symptoms through 21 categories that encompass symptoms and attitudes that describe behavioral, cognitive, affective and somatic manifestations of depression. Each item has four alternatives, with scores from zero to three. The score indicates absence of depression or minimal depressive symptoms (up to nine points), mild to moderate depression (10 to 18 points), moderate to severe depression (19 to 29 points) and severe depression (30 to 63 points).

To assess the quality of life of individuals with PD, the Parkinson's Disease Questionnaire (PDQ-39) was used^(20)^. The PDQ-39 contains 39 items covering eight domains: mobility, activities of daily living, emotional well-being, stigma, social support, cognition, communication and bodily discomfort, which can be answered with five different response options: “never”, “sometimes”, “rarely”, “frequently” and “always”. The total score ranges from zero to 100 and the higher the score, the worse the individual’s perception of their quality of life.

Data were analyzed using SPSS v.25.0.0 software (Chicago: SPSS Inc). Continuous variables were described as mean, standard deviation, median, and minimum and maximum values. Categorical variables were described as absolute and relative frequencies. Data distribution was verified using the Shapiro Wilk test. The correlation between the PDQ-39 communication domain and continuous variables was tested using Spearman's correlation test, and associations of this same domain and those between categorical variables were tested using Kruskal-Wallis test. Multiple linear regression models (stepwise entry method) were applied to understand which variables were most associated with variability in the PDQ-39-communication. First, two models were independently composed including the variables that presented statistical significance in the Spearman correlation test, since, due to the sample size, it would not be feasible to include all variables in a single model. Finally, a third model was created including the variables that presented significant association in the first two models. The significance level used was 5%.

## RESULTS

The final sample of this study consisted of 34 participants. Table 1 shows the sociodemographic and clinical characteristics.

**Table 1:** Sociodemographic and clinical characteristics of the sample

	**N (%)**	**Mean (SD)**	**Median (minimum - maximum)**
Total sample	34 (100)	-	-
Sex (M)	21 (61.76)	-	-
Age*	-	62.42 (12.21)	62 (40 – 85)
Educationarity*	-	7.25 (3.94)	6 (0 – 15)
Age of onset of symptoms*	-	55.36 (12.85)	57 (35 – 83)
Time of disease*	-	7.18 (3.95)	6 (2 – 17)
Levodopa equivalent dose	-	917.42 (463.51)	800 (200 - 2350)
**Initial Symptoms**			
Tremor	21 (61.76)	-	-
Rigidity	5 (14.70)	-	-
Bradykinesia	8 (23.54)	-	-
**UPDRS**			
NMADLE	-	10.06 (6.13)	9 (0 – 21)
MADLE	-	16.00 (7.14)	16 (3 – 37)
Motor examination	-	37.37 (12.36)	37 (12 – 59)
Motor complications	-	4.69 (3.74)	5 (0 – 12)
Total	-	66.97 (20.64)	66 (29 – 118)
**Hoehn & Yahr Scale**		2.12 (0.40)	2 (2 – 4)
2	28 (82.4)	-	-
2,5	3 (8.8)	-	-
3	1 (2.9)	-	-
4	2 (5.9)	-	-
**Cognition**			
MoCA	-	21.09 (4.27)	22 (11 – 27)
**Beck Inventory**			
Depression	-	12.42 (8.16)	11 (3 – 34)
Anxiety	-	17 (11.22)	13 (3 - 44)
**PDQ-39**			
Mobility	-	35.29 (21.23)	30 (0 – 85)
Activities of Daily Living	-	36.27 (24.63)	33.33 (0 – 79.16)
Emotional well-being	-	32.10 (18.98)	29.16 (0 – 66.66)
Stigma	-	29.41 (29.70)	31.25 (0 – 93.75)
Social Support	-	19.12 (21.47)	8.33 (0 – 66.66)
Cognition	-	30.15 (21.84)	25 (0 – 62.50)
Communication	-	20.09 (17.78)	16.66 (0 – 58.33)
Body Discomfort	-	42.40 (20.66)	50.00 (0 – 75.00)
Total	-	31.88 (15.72)	28.20 (5,12 – 62.82)

* in years

**Caption:** N = number of participants; SD = standard deviation; NMADLE = Non-motor aspects of daily life experiences; MADLE = Motor aspects of daily life experiences; UPDRS = Unified Parkinson's disease Rating Scale; MoCa = Montreal Cognitive Assessment; PDQ-39 = Parkinson's Disease Questionnaire

Table 2 presents the correlation between the PDQ-39 communication item and the continuous variables. Our study observed a positive correlation, meaning a connection where one variable changes alongside another. In this case, increases in the PDQ-39 communication item score were associated with an increase in disease duration, levels of depression and anxiety, difficulties in activities of daily living, emotional well-being, stigma, social support, cognition, body discomfort, disease stage (Hoehn & Yahr), as well as worse motor and non-motor symptoms, and total PDQ-39 scores. These findings suggest that, as PD progresses - with longer disease duration, worsening motor symptoms, and the emergence of non-motor symptoms (such as emotional and cognitive issues) - communication difficulties also become more pronounced, contributing to a further decline in overall quality of life.

**Table 2:** Correlation between the PDQ39 communication item and continuous variables

**Parameters**	**p**	**Correlation coefficient**
Age	0.37	-0.16
Education*	0.74	0.06
Age of onset of symptoms	0.13	-0.27
Time of disease	0.04*	0.36
Levodopa equivalent dose	0.08	0.31
**UPDRS**		
NMADLE	<0.01*	0.69
MADLE	<0.01*	0.62
Motor examination	0.11	0.29
Motor complications	<0.01*	0.48
Total	<0.01*	0.61
Hoehn & Yahr Scale	<0.01*	0.51
**Cognition**		
MoCA	0.47	-0.13
**Beck Inventory**		
Depression	0.03*	0.37
Anxiety	<0.01*	0.56
**PDQ-39**		
Mobility	0.16	0.25
Activities of Daily Living	<0.01*	0.66
Emotional well-being	<0.01*	0.62
Stigma	0.02*	0.4
Social Support	0.02*	0.39
Cognition	0.02*	0.41
Body Discomfort	<0.01*	0.47
Total	<0.01*	0.75

Correlation Spearman’s test

* p<0,05

**Caption:** NMADLE = Non-motor aspects of daily life experiences; MADLE = Motor aspects of daily life experiences; UPDRS = Unified Parkinson's disease Rating Scale; MoCa = Montreal Cognitive Assessment; PDQ-39 = Parkinson's Disease Questionnaire

Regarding the categorical variables, there was no significant difference between the PDQ-39 communication item and the gender of the participants (p=0.70). However, as observed in Table 3, there was a difference in relation to the initial symptom (p=0.01). Individuals with rigidity as an initial symptom had a higher score on the communication item of the PDQ-39 when compared to those who started the disease with tremor.

**Table 3:** Comparisons between initial PD symptoms and the PDQ39-Communication item score

**Initial Symptoms**	**PDQ-39-Communication - Mean (SD)**	**p (post-hoc test)**
Tremor	12.50 (13.38)	Tremor X Bradykinesia: 0.206
Rigidity	35.00 (14.91)	Rigidity X Tremor: 0.029*
Bradykinesia	26.04 (19.64)	Bradykinesia X Rigidity: 1.000

Kruskal-Wallis test and Bonferrini post-hoc test

* p<0.05

**Caption:** PDQ-39 = Parkinson's Disease Questionnaire

Table 4 shows the result of the multiple linear regression (stepwise entry method). Two analytical models were developed to better understand which factors influenced communication-related quality of life in individuals. The first model included variables such as disease duration, depression, anxiety, non-motor aspects of daily life experiences, motor aspects of daily life experiences, and motor complications. The second model included emotional well-being, stigma, social support, cognition, and bodily discomfort. From these two models, only the variables that showed statistical significance were included in the final model (activities of daily living, emotional well-being, motor and non-motor aspects of life experience).

**Table 4:** Multiple linear regression with the dependent variable PDQ-39-communication

Independent variable	β	p
PDQ-39 – activities of daily living	0.30	0.006*
UPDRS - non-motor aspects of life experience	1.28	0.007*

Multiple linear regression model (stepwise entry method)

* p<0.05

Note: general regression results R: 0.75; R^2^: 0.56; F: 18.77

**Caption:** β = non-standardized coefficient; PDQ-39 = Parkinson's Disease Questionnaire; UPDRS = Unified Parkinson's disease Rating Scale

The final model demonstrated that greater variations in daily activities and non-motor aspects (such as emotional and cognitive problems) were associated with greater changes in communication-related quality of life. These results highlight that the most significant predictors of communication-related quality of life in PD were variations in daily activities and non-motor aspects of daily life, suggesting that impairments in these areas have a substantial impact on individuals' ability to communicate effectively.

## DISCUSSION

To the best of our knowledge, this is the first study that investigated the correlation between the quality of life in communication of people with PD and clinical and sociodemographic variables in Brazil.

Through the multiple regression models performed in our study, we can observe that the main predictors of quality of life in communication are activities of daily living (PDQ-39) and non-motor aspects of daily life experience (UPDRS), and the final regression model shows that these variables together are capable of explaining 56% of the variance in PDQ39-communication. The results indicated that for each one point increase in PDQ-39 - activities of daily living, there is an increase of 0.30 points in PDQ-39-communication. In other words, those with a worse perception of the performance of their daily activities have a worse quality of life in communication.

Another variable that was significant in the multiple regression showed that for each point increase in the item non-motor aspects of daily life experience of the UPDRS, there is an increase of 1.28 points in the item communication of the PDQ39. With this, it is possible to understand that the motor aspects are not predictors of quality of life in communication, contrary to what one might think, due to the fact that patients with PD have motor speech disorders. The impact of non-motor symptoms on the quality of life of individuals with PD has attracted attention, some studies have reported that non-motor symptoms play a more important role in the decline in quality of life than motor symptoms^(21-23)^.

In our analyses, multiple regression was the most important test and indicated the main predictors of quality of life in communication. The correlations studied also indicated interesting relationships between quality of life in communication and other variables, however such variables cannot be considered predictors of quality of life in communication, but rather variables that are associated in some way.

In our study, there was correlation between quality of life in communication and disease duration, Beck scale (depression and anxiety), PDQ-39 (activities of daily living, emotional well-being, stigma, social support, cognition, bodily discomfort and total), Hoehn & Yahr scale and UPDRS (non-motor aspects of daily living experiences, motor aspects of daily living experiences, motor complications and total). Previous studies report that complaints regarding communication are correlated with quality of life in patients with PD^(8,24-26)^. Just as the literature indicates that the quality of life of people with PD is lower when bradykinesia, rigidity, freezing of gait, depression, fatigue, cognitive decline and sleep disorders are present^(25-27)^.

Considering our findings and the literature, impairment of activities of daily living leads to social isolation, depression and anxiety, which compromises communication^(26,28)^. However, impaired ability to communicate also leads to greater isolation in a disease that favors social isolation. Previous studies have shown that communication is a significant concern for people with PD because it limits their social relationships^(29)^.

Our study also showed that individuals who presented with rigidity as an initial symptom had significantly higher scores on the PDQ-39 communication item than those individuals who started with tremor. The literature explains that muscle rigidity in PD is one of the most common motor symptoms and results in increased involuntary muscle activity^(30)^. The literature also shows that rigidity has a significant relationship with speech and phonation due to the way the disease affects the muscles involved in the motor act of speech and vocal production^(31)^. These combined factors result in speech that may be more difficult to understand, which may affects the communication of patients with PD, thus influencing the functionality of communication and consequently affecting the quality of life in communication^(30-33)^.

Despite the complexity of PD, there is a dearth of literature addressing quality of life and communication. In other neurodegenerative diseases, such as Amyotrophic Lateral Sclerosis (ALS), Alzheimer's Disease (AD), and Machado-Joseph Disease, there are studies exploring quality of life^(34-41)^. However, there is also a dearth of research that specifically focuses on quality of life in the context of communication. It is important to mention that some studies indicate that, in both ALS and AD, non-motor symptoms also play a significant role in quality of life^(36,38,39)^.

The interpretation of the results of this study should consider the limitation that quality of life in terms of communication was not assessed through a specific protocol focused on this aspect, but rather through an item inserted in a global quality of life protocol. Although there are specific scales and questionnaires to assess communication problems in patients, generally answered by the individual themselves or by their caregiver/companion, there are few assessment measures specifically designed to identify difficulties in the use of communication in the daily life of patients with PD. Another significant limitation was the absence of an evaluation of the communication of the participants, which could have provided more nuanced insights into how these issues impact their overall quality of life and social interactions. Therefore, further studies are necessary to address these gaps, focusing not only on the development of more accurate assessment tools but also on exploring the multidimensional nature of communication challenges faced by patients with PD.

## CONCLUSION

Communication in individuals with PD is affected by multiple factors related to disease progression. Notably, activities of daily living and non-motor aspects play a key role in influencing communication-related quality of life. These findings contribute to clinical management by emphasizing the importance of addressing non-motor symptoms in treatment plans, focusing on improving quality of life beyond motor symptoms.
